# Accessibility of intimate partner violence-related services for young women in Spain. Qualitative study on professionals’ perspectives

**DOI:** 10.1371/journal.pone.0297886

**Published:** 2024-04-04

**Authors:** Laura Otero-García, Eva Durán-Martín, Esther Castellanos-Torres, Belén Sanz-Barbero, Carmen Vives-Cases

**Affiliations:** 1 Nursing Department, Faculty of Medicine, Universidad Autónoma de Madrid (UAM), Madrid, Spain; 2 CIBER of Epidemiology and Public Health (CIBERESP), Madrid, Spain; 3 National School of Public Health, Instituto de Salud Carlos III, Madrid, Spain; 4 International Doctoral School of the Universidad Nacional de Educación a Distancia and the Joint Research Institute of the Nacional School of Health (UNED- IMIENS), Madrid, Spain; 5 Public Health Research Group, Department of Community Nursing, Preventive Medicine and Public Health an History of Science, University of Alicante, Alicante, Spain; 6 Department of Social Sciences, University Carlos III, Madrid, Spain; Drexel University, UNITED STATES

## Abstract

**Introduction:**

Intimate partner violence (IPV) is common among young people, but the use of IPV resources among young adult women and teenagers is limited. This study aims to analyze professionals’ perceptions about the main barriers and facilitators encountered by young women (16–29 years old) exposed to intimate partner violence (IPV) when accessing formal services in Spain.

**Methods:**

Qualitative study based on 17 in depth interviews carried out in 2019 with professionals who manage resources for IPV care in Madrid (Spain) from different sectors (social services, health care, security forces, women or youth issues offices, associations). A qualitative content analysis was conducted.

**Results:**

The professionals interviewed perceive the following barriers: 1) Time it takes for young women to recognize IPV because the social construction of sexual-affective relationships is permeated by gender inequality; 2) The process of leaving a situation of abuse; 3) Barriers inherent to IPV services. The key aspects to improve access to these resources are related to care services, professional practice, and the young women themselves.

**Conclusions:**

There are both psychosocial barriers, derived from the process of leaving a situation of violence, as well as structural barriers for young women to access and properly use the recognized services specifically aimed at them or comprehensive IPV care. Services need to be tailored to the needs of young women so they can be truly effective in order to escape IPV.

## Introduction

Intimate partner violence (IPV) is recognized by the World Health Organization (WHO) as one of the most common forms of violence against women. It includes behaviors from an intimate partner or ex-partner that cause physical, sexual and/or psychological harm, including physical aggression, emotional abuse, control and economic IPV practices and sexual coercion [1]. IPV and/or sexual violence is considered a public health problem that has short, medium, and long-term impacts on the physical and mental health of women exposed to this type of violence [2–6]. It also has negative effects on children’s physical and mental well-being [1]. Strategies should be found to improve access to services for young women experiencing IPV and provide them with the skills and support to leave violent relationships, thus preventing its consequences [7].

In recent years, dating violence among teens and young people has been the focus of research both in Spain and all over the world [8–11], and differences have been found between teen dating violence and IPV occurring in adult couples. The data in Spain reflect a higher prevalence of physical and sexual IPV in young women (aged 16–29) compared to adults (aged 30–49) [12]. In addition, within the types of psychological violence, controlling violence is the most reported by young women when compared to adults and older women (aged over 50). However, adult and older women experience psychological and emotional violence more often than younger women [12].

In Spain, there are specialised and non-specialised IPV services which are delivered both in specialist IPV units and community health services. Different care services have been made available, such as: police and security assistance, legal assistance and guidance, comprehensive health and social care, both in primary care and in specialized care, as well as other services provided by feminist associations and women’s care centers [13, 14]. A cross-sectional study shows that formal services have been used to a lesser extent by young women, in this case only 41% when compared to 51% and 48% by adult or older women, respectively. Formal psychology/psychiatry services were the most used by all women, young (27.4%), adult (33.6%) and older women (29.3%). However, legal and/or social services were the least used by young women, 9–10% compared to 12–19% in adult and older women, respectively [15]. Previously published studies show how young women face a series of barriers in accessing formal services, which could explain the aforementioned data. In Spain, these barriers include the normalisation among young people of violence within dating relationships, and the limited training of professionals to deal with this problem in young women [16–19]. Other international studies also reflect a lack of training, as well as awareness of IPV among professionals, and highlight the limited information on available resources [20, 21]. Furthermore, a study in Sweden reflects the barriers caused by the lack of adaptation of resources to young people [21].

In a similar sense to what is observed in young women, those who face IPV in other vulnerable contexts also suffer from differences in accessing resources. Older women with a low socioeconomic status seek fewer formal services and report violence less. Some studies show that this may be due to older women being more economically dependent [15, 22]. In Spain, young and old immigrant women, as well as Roma women, also use formal services less frequently [23, 24]. In a mixed-method study, women with disabilities barely use formal resources at all due to the lack of universal access, as they are not adapted to their own needs. This also occurs in other areas in a society marked by ableism [25].

Previous articles highlight the scarcity of studies regarding access to resources for young women victims of IPV. Taking into account the perspective of professionals’ who work in these kinds of services, allows us to assess the situation of young women and their difficulties in accessing these services. Thus, this study aims to analyze professionals’ perceptions about the main barriers and facilitators encountered by young women (16–29 years old) exposed to IPV when accessing formal services in Spain.

## Methods

### Study design

This study is part of the research project “Intimate partner violence in young people in Spain: Epidemiology, impact on health, accessibility and use of health and social care services for IPV”. The study was carried out in the Region of Madrid in 2019 and has a qualitative design using semi-structured interviews with 17 professionals. The COREQ Checklist for reporting qualitative research was used and is attached as supporting information (S1 Table).

In order to design a heterogeneous sample, the authors ECT and BSB established the following criteria. Firstly, the interviewees needed to be professionals in the field of IPV intervention. Secondly, selected professionals worked in different care areas (nursing, social work, psychology, police forces, public administration workers, etc), and thirdly, they belonged to different administration levels (state, regional, municipal, community) so that the sample had a variety of administrations levels represented.

Purposeful sampling was used, identifying a series of key informants. The strategy for the identification, selection and recruitment of participants was as follows: ECT proposed the selection of participants based on their contacts in the community and professional field as an equality agent, which was discussed among all the authors and new potential informants were included to complete the sample. [Equality agent is a qualified professional figure in the field of equality policies and violence against women]. ECT first contacted the 17 potential professionals by telephone, and when they agreed to participate in the study they were formally invited via an email. This email contextualized the study and explained the aim of the interview. All professionals finally accepted to participate. Once they agreed to participate, a face-to-face individual interview was conducted by the interviewer in the workplace of the interviewees. In one exceptional case, two professionals with ECT were present in the interview. The 17 professionals (14 women and 3 men) had a varied background and professional experience and included nurses, a psychiatrist, professionals from the state security forces, psychologists, social workers and those responsible for youth centers or gender violence. These professionals worked at one of the four administrative levels required (state, regional, municipal and community).

### Data collection

Before conducting the semi-structured interviews, the interview script was drafted to include topics related to the perception of the current situation of IPV in young women, its approach from the professional practice perspective, difficulties and facilitators in accessing IPV resources and proposals for improvement (see S2 Table). In this study, when we refer to young women, they are those aged 16–29 years old who have been previously identified as belonging to a violence-related risk group in our previous study [12]. This script was elaborated and discussed by all authors before being validated by the research team. A pilot test was not conducted, but the first two interviews were used to check fluidity among the questions in each section, and the results from this check were taken into account for the remaining interviews. All interviews were audio recorded and field notes were taken. In addition, the interviewer (ECT) also recorded her experience and perceptions of the interview. The interviews lasted between 45 and 90 minutes, with an average of 1 hour. One interview had to be repeated due to a problem with the recording. When the last interviews did not generate additional information, it was considered that data saturation had been reached. The field work was carried out between March and July 2019.

### Ethical considerations

Each professional was given an informed consent form, where the objectives of the study were explained, as well as the reasons for the interview. Their anonymity and confidentiality of the opinions they expressed was guaranteed and all participants signed a consent form. Furthermore, they were assured that their participation was voluntary and that they could withdraw at any time during the interviews without having to provide any explanation. The project was approved by the Ethics Committee of the Carlos III Health Institute (CEIPI612020-v3).

### Data analysis

Once all the information was collected, each interview was transcribed verbatim. After reading each interview, ECT carried out a pre-analysis (with support from LOG) to organize the information according to the different dimensions of the interview script (related to the objectives of the research) and identify patterns. ECT then gave an initial coding to each interview’s dimensions based on a detailed reading, following the qualitative content analysis approach [26, 27]. The codes were then grouped into categories according to similarities, following the inductive approach of content analysis. The initial results were refined until completed and these findings were placed in the context of similar literature. This was done by the rest of the authors (LOG, BSB, EDM, CVC) In this way the research data was properly triangulated.

### Reflexivity

The team members have experience in qualitative in IPV, public health and gender research. This experience may influence in theoretical frameworks of this study in the factors influencing women’s access to IPV-related services. These factors have been previously applied to the topic of gender inequalities in the access to health services. Moreover, this experience was important for the study design, sample selection and data collection. In order to ensure trustworthiness, the results were critically reviewed by all authors, verbatim quotations were used and data saturation was reached. In addition, a qualitative content analysis was conducted because of its inductive approach, i.e., it is based on the data, and allowed the impact of the researchers on the results to be minimized. This facilitated categories to be formed from the similarity between codes, rather than fitting the data to predefined patterns or themes, or a pre-established theorical model.

## Results

The professionals (see Table 1) have identified three types of barriers that affect young women and hinder their access to formal resources related to IPV. On the other hand, the professionals identify certain key aspects that could improve these barriers.

**Table 1:** Characteristics of the professionals interviewed.

Number of interviews	Sex	Area or professional profile		ID	Scope of action
2	M	Professionals of state security forces in charge of the IPV Surveillance Register at police level.	Psychology	MP12	State
			Master’s in Criminology. Specialist in Forensic Psychology		
			Social Work	MP16	
2	F	Nurses	Responsible for IPV in a health center	WP13	Regional/ Municipal
			Management in a health center	WP3	Municipal
1	F	Psychiatrist in a mental health center		WP10	Regional/ Municipal
3	F	Psychologists	in a specific service of sexual violence	WP15	Regional/ Community
			in a specific service of youth violence	WP14	Regional/ Community
			Psychologist of a feminist association	WP7	Community
1	F	Social worker and psychologist in a specific IPV center.		WP4	Municipal
4	F	Social workers	in a specific IPV center and social anthropology trained	WP9	Regional / Community
				WP5	Municipal
			in a local corporation	WP1	Municipal
			in a mental health center	WP11	Regional/ Municipal
2	F	Responsible for the youth area in a local corporation		WP2	Municipal
				WP6	Municipal
1	F	Director of an entity related to IPV in the tertiary sector trained in psychology and social work.		WP8	Community

F = Female. M = Male; MP = Professional man. WP = Professional Woman.

### Barriers related to the gender socialization of young women and the process of leaving a situation of violence

#### A) Barriers related to the socialization process of women in gender

In Spain, professionals state that normalizing violence in intimate relationships is based on gender socialization. In this sense, they believe there are certain practices associated with control and/or emotional psychological violence that young women do not identify as IPV. There are myths about love that are harmful to couples, but are normalized in music, films and television series, for example, expressions of “love” that justify jealousy, emotional dependence, lack of autonomy, etc. Professionals report that this legitimization of IPV is reinforced by the illusion of current equality, in which, despite the formal equality of rights for women and men, inequality in daily practices continues to exist. For instance, they may think that IPV is something from the past, when the women had fewer rights and were dependent on their husbands. Now that they have “equality” and are independent women, it cannot happen to them.

“*Violence has been normalized in daily relationships (…) the music they listen to*, *the series they watch*, *Twilight… This doesn´t help at all to detect situations that put them at risk*” (MP16).

“*They think it´s not going to happen to them or that they´re in a situation of equality and boys aren´t like that anymore*. *[…] Thinking it won´t happen to me is one of the irrational ideas*” (WP7).

Health care professionals declare that only when there is physical abuse do young women clearly identify it as IPV, and this is when they seek help.

“*Most of the times they access the services when there is significant physical violence*. *Women come with physical injuries or with severe anxiety crises because they have been physically abused*” (WP13).

Likewise, one of the social worker’s interviewed reports that there is a social denial of IPV. This denial is cultural and is associated with a patriarchal ideology where violence is an individual issue, not a structural one, and thus, should be treated as a “couple´s conflict”. In other words, it is a problem created by some women and does not exist or affect society. This barrier of ideological nature, or occurring as a result of a patriarchal reaction, makes identifying violence itself a problem, as some behaviours are socially accepted.

*“(…) we also have an ideological barrier in our work (…) this is something four crazy women want to bring up a as a subject that isn´t a problem*, *that isn´t real [irony]*. *This is what’s said on the street*, *and it generates a lot of friction in our interventions*. *It’s difficult to make them understand listen*, *what’s happening to your daughter is real (…)*” (WP5).

#### B) Barriers derived from the process itself of leaving a situation of IPV

From the point of view of the interviewed professionals, young women also face different fears derived from the process of leaving a situation of violence. First of all, they do not know what resources are available, or what the established protocols are in this sense. Secondly, they are frightened about the possible response of their families, loneliness, and loss of friends. They fear rejection, stigmatization or that people will believe they are not telling the truth. In addition, the process of leaving will depend on the type of violence the young woman has suffered. For example, physical violence is easier to identify and seek help for than psychological violence. In this sense, the professionals point out that, in a situation of sexual violence, young women do not know where to go or what they will find:

“*They don´t know about any resources*, *they have run out of friends; my parents are going to find out (…)*, *Well*, *and if it’s a beating*, *it’s an examination*, *file a complaint with the police*, *with the doctor when he explores her* [physical examination]. *If there’s been a sexual assault*, *collection of samples*, *notify the coroner*. [In Spain, laboratory samples and evidence for the judicial process must be collected by a coroner and sent to the court. Therefore, gynecologists and coroners are involved]. *Access the resources (…) above all*, *it´s ignorance because they don´t know what they have to do*” (WP11).

“*Fear*, *eh (…) because of what they´re going to find*, *they think that they may have to report it (…)*. *It’s an optional thing*, *that we´ll respect their decision there are lots of people who don´t call because they aren´t sure*” (WP6).

Another perceived barrier is the psycho-emotional situation of young women and their ability to talk about the situation of violence and leave it.

*“And obviously their own barriers*, *their defences*, *that the girl feels it’s really the right time to get help and propose a therapeutic process”* (WP14).

For psychologists, another barrier inherent to young underage girls is their legal situation. As they are minors, under 18 years of age, they depend on their parents´ authorization or, in their absence, of their guardians, to use the service:

“*I’m 14 years old*, *I want to start therapy*, *my mother completely agrees but my father doesn´t sign to allow it (…)*, *the difficulty of having to have an authorization is there” (WP15)*.

*In the case of teenagers under the age of 16*, *the signature from both parents is required in order to receive care*” (WP14).

### Barriers related to professionals working in resources against IPV

Another of the difficulties that informants find hinder access to these services is the lack of detection due to insufficient specific professional training, both in equality, gender perspective and specifically in IPV in adolescent and young women.

“*Training in violence for health professionals is scarce and*, *above all*, *there’s no training on gender perspective*, *nor on equality or training on access to resources for women to which they can be referred*” (WP13).

In addition, stereotypes play an important role in professionals who are working with young and adolescent women. Some believe and perpetuate these stereotypes, observing a certain permissiveness regarding conflictive situations related to the developmental period of adolescence and youth.

“*I think it is minimized a lot in young girls (…)*. *you´re still very young (…) adolescents are like that*, *it´s known that boys drink alcohol and you know… first relationships aren’t very stable (…) from the age of 13*, *well*, *come on*… *he´s been a rebel (*…*) when he´s 18*, *it´ll be over*… *and so*, *the violence we see there*, *especially sexual violence from the age of 14 when they begin relationships until the age of 16–18*, *is worked on very little or not at all”* (WP13).

Some professionals believe training in gender issues is not valued. Specifically, there are hardly any professionals with specific training to address sexual violence. From the point of view of the professionals, this means that young women who wish to get help and use the resources available do not receive quality care, and are even sometimes questioned by the professionals, thus leading to secondary victimization.

“*Sexual violence*, *this isn’t discussed and there are no trained professionals*. *So*, *well*, *if you’re not trained*, *you’re encouraging this to continue*. *Reactions of*: *"Well*, *girl*, *it wasn´t so bad because there was no penetration" or very revictimizing situations such as*: *"Hire a detective" [private detective to investigate the victim after the assault to see if they are getting on with their life] or the fright of sexual violence that generates fear*, *generates doubts*, *horror*, *disgust*, *for this first denial of “it can’t be happening to me” but not only to the victim*, *but also to those who listen”* (WP15).

This secondary victimization can cause them anxiety and may even lead them to question their decision to get away from the violence.

*“… you have to keep telling the story*, *to each professional you go to*, *repeat the same thing over and over again*. *So*, *many times*, *when thinking about it or acting you have to take this into account”* (WP11).

### Structural barriers within the resources available to tackle IPV against women

Structural barriers identified by practitioners will be presented (see Table 2).

**Table 2:** Structural resource barriers to respond to young women experiencing IPV.

Structural resource barriers
• Services not adapted to the young age group. • Non-identification with IPV “victim” resources. • Lack of intersectionality in attention (migrant women, young women, disabled women, other ethnic groups, etc.). • Unattractive and confusing locations of IPV resources for young people (e.g. social services). • Lack of availability of specialized IPV services for young people. • Problems of geographic access. • Lack of confidentiality of services in rural areas. • Lack of diffusion and real contact of IPV services with young people. • Limited human, material and financial resources. • Unfriendly and insecure spaces.

One barrier identified by the professionals in a specific service of youth violence is that there are no IPV resources adapted to young women. The perception of the interviewees is that the services are designed for adult women. Young women seek these resources because they are pushed to do so by their relatives, friends or because a complaint has already been filed.

*“The question is whether they feel identified or not [with the adult battered women who come to the services]*. *Hey*, *some of the girls who come through here come from care services for adult women*, *where they haven´t felt so identified enough [to seek help and support independently to meet their needs following the violence they have suffered]*” (WP14).

Likewise, the professionals point out that the name of the service itself can alienate young women.

“*In other words*, *the structure of the service itself is not designed for young people (…)*. *And on top of that*, *the name should also be looked at because it doesn’t help (…) there are people who don’t want to go to a place with the word victim in the name*” (WP9).

In addition, they are designed with a very specific user-care profile in mind, which is one of a middle-aged woman. Thus, it does not consider the intersectionality of other situations of oppression or discrimination women are experiencing.

“*Formal resources aren’t adapted to anyone who falls outside the average Spanish woman category (*…*) older women don´t come*, *women with mental health problems don´t come*, *other women with whatever don´t come*… *or when they do*, *they aren´t treated because the services aren´t prepared (*…*) intersectionality is lacking*. *Resources are not ready*” (WP9).

At the same time, professionals mention that most IPV resources are found within social services, which is far from what a young girl imagines is the type of help she needs. There is a traditional image of what social care interventions are, but this image is far from the type of work carried out for IPV.

“*They basically don’t go*, *in general*, *because the municipal services aren’t normally located next to the youth centres*, *the usual thing is a social services centre*, *which makes it very difficult*, *so to speak*, *to give a friendly image to young girls*” (WP8).

On the other hand, in small municipalities (in rural areas) social services are found together with other municipal services. This can lead to people not using them, as the confidentiality of those who go to the IPV help desk is lost when they are seen in the waiting rooms of the other services:

“*We are in 7 towns …*. *Sometimes we’re seeing people in the City Hall and the local police door is next door*. *This must be considered*. *Maybe you don’t realize when you arrive and sit at the table*, *but when you look around you say to yourself*: *of course*, *I wouldn´t go in*, *I wouldn´t go in with this situation [victim of IPV] because I just want information*” (WP5).

Likewise, one of the informants, who comes from the health field, indicates there is a lack of real contact with youths, as well as knowledge on the resources they can use.

“*I think there’s a lack of contact with the services*. *Come on*, *we’re talking about fundamentally young people together with the rest of the formal and non-formal resources of the network; that is*, *youth associations*. *You must go out*, *you must make yourself known to facilitate the contact*, *so the girls themselves can see you*” (WP10).

Another of the barriers identified are the problems of accessibility to specialized services for young women and adolescents who live outside the municipality of Madrid and/or who also live in rural area municipalities. This perception depends, to a large degree, on the distance between the municipality they live in and where the service is located, the available means of public transport and the economic capacity of the women and their families. [Currently, the Region of Madrid, where the study is focused, has a single specific service for young people who have suffered sexual violence. However, in August 2021, another one was announced, but it is not yet underway. Both will be located in the city of Madrid.] This can be an access barrier for young women who do not live in the city of Madrid, who need to have, on the one hand, the time to go to the service and, on the other hand, the economic capacity to be able to move around.

“*The economic cost*, *the time*, *missing lessons (…) if you go to a service in Madrid and you have to be there at 2*, *3 or 6 in the afternoon*, *(…) you have to leave your house very early*. *Afterwards*, *you may not go back to the second appointment because of course*, *I have to get there*, *(…) see a stranger*, *because the bond has to be formed and for a long time*. *So*, *it isn´t worth the effort of making the trip to work on it*” (WP5).

The services of the municipalities adjacent to large urban centres do not have the level of specialization that the specific adolescent program has. In other municipalities of the Region of Madrid, there is indeed a general need for specific centres for adolescents.

“*They´re usually offered care from their municipality services*, *but of course*, *they don´t have the level of specialization and specificity that we have as a resource for adolescents*” (WP14).

Along these same lines, regarding the structural conditions of the services that limit or pose a barrier to young women´s access is the lack of professionals with specific training, particularly psychologists, to care for young women and adolescents. Even so, these professionals do everything possible to see to them. However, for some professionals, the lack of trained human resources is one of the barriers that, at the same time, makes it difficult to professionally deal with young women and adolescents.

“*In general*, *we treat adult women; we don´t have a child psychologist*. *It´s true that*, *due to the circumstances of the municipality and that the main services to care for adolescents are in Madrid*, *(…) they end up coming to us or to the children’s mental health services (…) we see them partly so they aren´t abandoned*, *but we really shouldn´t be the ones to deal with them*” (WP4).

Professionals complain about the lack of personnel and the fact that the budget allocated is not enough for the prevention and care of IPV and this becomes a structural barrier.

“*The budget*, *the staff*. *In other words*, *it´s always the same*, *we don´t have money for many things we want to start*, *we’re the same people*, *we want to do things*, *but if we do more*, *we have to stop doing other things*” (WP6).

On the other hand, police informants point out that the police infrastructure where women are seen is not the most adequate. There is a lack of privacy, and a physical space is needed that generates complicity and trust to be able to recount the events.

“*I wish we had the possibility of talking to the victims in a closed office*. *During these years*, *I´ve managed complaints where I have had the victim here and*, *behind them*, *there was another boy picking up a complaint for loss of ID*, *robbery by force*, *or sometimes people or arrestees just walking behind*… *a disaster*” (MP16).

### Key aspects to improve accessibility to resources

#### A) Facilitators of women care services to serve young women experiencing IPV

In order to facilitate young women´s access to care services and improve detecting cases, some professionals carry out IPV awareness raising activities to enhance mental health literacy around the issue and inform about resources available to young women. In addition, they participate in events organized around key days such as 8^th^ March, International Women’s Day, and 25^th^ November, International Day for the Elimination of Violence against Women. They also point out that the work done with sexuality and emotional relationship interventions from non-specific violence resources, such as family planning services, youth centres focusing on affective and sexual education, colleges etc, fosters greater knowledge and access to resources.

“*All of us go to the school center once a month and they get to know us (…) In this way*, *we’re there and we generate the necessary climate of trust*, *closeness*, *and accessibility*” (WP3).

“*We participated in the key dates of 25*^*th*^ *November and 8*^*th*^ *March (…) it’s a real pleasure to see that there are so many young people*, *loads*, *and we see more and more every time”* (WP2).

“*They come to our service for family planning (…) when we carry out our intervention*, *we’re aware of IPV situations*” (WP3).

Time flexibility, as well as the availability of safe, accessible and youth-friendly spaces are also considered facilitators. On the other hand, the coordination between educational centres, social services, violence help desks and counselling departments are perceived by the informants to be fundamental in providing adequate care to young women. Specific and coordination protocols amongst all the services that attend victims are needed to guarantee such coordination. Finally, it also helps young people to come to this type of service if community movements are involved in society projects. These facilitators have been shown to be effective in some services, as indicated by professionals below:

“*Having a schedule accessible to them and a more attractive image (…)*. *This change of location is also making it more accessible (…)*, *it’s bringing us more new people*” (WP6).

“*In each site we organize ourselves differently (…) There isn´t an institutional strategy*, *but it’s direct because she has an interest* [the social worker] *and so do we”* (WP4).

*“The fact that the people of your neighbourhood are involved*, *I believe that helps to introduce the issue of IPV as something that’s transversal (*…*) we all have to be there*, *in everyday life*” (WP10).

#### B) Facilitators related to the general practice of serving young women experiencing IPV

One of the factors that has made it easier for professionals to provide adequate care to young women is having training on gender perspective, as well as on adolescent-tailored training. On the other hand, professionals must respond to situations as they arise and give quality solutions to young women. However, in many cases professionals must spend their free time providing these adequate solutions, something that is not recognized at an institutional level.

“*I’m trained in group work*, *managing complicated adolescents*, *gender (…) being aware of my own career*, *… it has helped me work with boys and girls to prevent IPV*” (WP3).

“*Personal involvement is crucial*. *You must put your heart into it*. *They’re certainly not going to pay you for it*” (WP14).

Thus, it is also a facilitating element identified by most informants if professionals have the necessary experience in multidisciplinary and specialized work. In addition, professional performance using active listening, empathy and giving the feeling you believe them are some other key principles that enable better interaction. A good practice is not to show blame, but to generate a climate of trust that fosters the creation of a bond with the victim. Additionally, when professionals show they are available, making the girls feel they can come to them when needed, this facilitates care follow-up.

“*Empathy*, *the fact that they feel they’re believed*, *this is crucial*” (WP1).

“*We have to listen to them more carefully (…) they need to know they aren´t to blame*, *(*…*) with young girls*, *their tempo is different (…)*, *as soon as they feel better*, *they´ll stop coming*, *even if the process hasn´t ended …*. *And giving them this freedom and autonomy is also important*” (WP8).

Another of the adaptations professionals have made is to modify their language, image and communication channels through social networks in order to better reach young girls.

“*We’re adapting our language so they can get to know us*, *so they feel comfortable” (MP5)*.

*We’re making an investment from the moment we manage these networks in a professional manner*” (WP7).

Having a point of reference for IPV in primary care has helped to improve coordination.

“*Now in health care there’s a point of reference for IPV (…) you notice the change a lot (…) there’s more openness to coordinate this type of situations*” (WP8).

#### C) Improvement in the services dealing with IPV that originate from young girls themselves

For informants working in the management of these resources, peer-to-peer and self-managed groups have been shown to be effective. Women in these groups have helped others to access these resources. Finally, professionals consider it is effective if the young women who have faced IPV assume a leading and participative role, which leads to actions where both they and the young girls can be involved.

“*Girls who actively participate become a sort of reference for other girls in similar situations because they can help them from their point of view*” (WP8).

“*The young girls asked us for a workshop on self-defence and we did it*. *This was requested by them …”* (WP2).

## Discussion

The results of this research show there are three types of barriers for young women suffering IPV to access services: 1) Barriers related to young women´s differential socialization and process of leaving the situation of violence; 2) Barriers related to the professionals who work in the IPV services; 3) Structural barriers in the services themselves. The key aspects for improving access to IPV resources for young women will be discussed below, related to each of the barriers identified, in order to propose some solutions.

In short, the barriers found in this study are similar to those found in other studies addressing access to services by women and other vulnerable groups [20, 28, 29]. From a professional perspective in Spain, specifically from the health field, similar barriers arise, such as the lack of specific training in IPV in primary care, not being able to work in multidisciplinary teams or difficulties immigrant women find in accessing the services [18]. Law 1/2004 on integrated protection measures against IPV includes a clause that gives special consideration to women in vulnerable situations [30]. Despite this, professionals believe specific protocols adapted to each group are still insufficient. Nonetheless, similar barriers also exist in studies focusing on young people’s access to services in Sweden, for instance services that are not youth-friendly or not welcoming, and services that do not take into account the intersectionality of people [21]. Finally, the challenges here identified coincide with those found in other studies, in Spain and elsewhere, which highlight the importance of promoting support groups among young women, community awareness and coordination and support between different professions [18, 28]. Furthermore, a key aspect for improvement is the need to have action protocols, as when they are lacking it can be a hindrance [18, 28].

The most important barriers will be discussed below, together with their key areas for improvement.

Regarding the barriers related to the socialization process of young women and those derived from the process of leaving the situation of violence itself, it has been found that this type of violence is more normalized in this age group than in others due to false beliefs about how love relationships are built. Control and jealousy behaviours are normalized as signs of love, thus posing a barrier for young women to identify this type of violence and seek help [16, 31, 32]. On the other hand, an important barrier is the lack of knowledge about the resources available to leave the situation of violence. This could be due to media campaigns not being adequately adapted to the needs of young women [16]. Campaigns do not reflect the characteristics of IPV among young people [16], and the information does not reach the victim’s circle of friends and family. In order to solve this lack of knowledge, it is essential to be present in the educational environment, not only to prevent and detect violence, but to inform young people about the services available to help them in these situations. There is ample evidence about how implementing prevention programs increases knowledge about what women must do and where they can go for help if subjected to IPV [33–36]. In addition, such programs aimed at young boys could help reduce the occurrence of violence and, on the other hand, help young women detect violence earlier, thus reducing its consequences, that is, secondary and tertiary prevention. This study has identified some aspects that could help overcome these barriers, such as systematically being present in education centres and organizing activities on designated dates (8^th^ March, International Women’s Day and 25^th^ November, International Day for the Elimination of Gender Violence) which would increase knowledge and make young women more aware of the different IPV help services available to them. In addition, dealing with affective sexual issues of relationships in services not specifically designed to treat violence enables young women to recognize signs of violence more easily and, therefore, seek help [37]. Other barriers, both related to each other, are those concerning the cultural denial of IPV, and the fear of the reaction caused within their social environment. These barriers in young women could be due to several reasons. On the one hand, the false belief in gender equality that can lead to such denial [16]. A European study also reflects on the discordance in Nordic countries. Surprising as it may seem, they have the highest gender equality indices and the highest prevalence of IPV in the general population [38]. This is called the “Nordic paradox” for which various explanations have been attempted. One of them is the under-declaration related to a false belief in gender equality as it makes IPV increasingly confined to the private sphere, where women are perceived as independent and self-sufficient to fight against it [39]. On the other hand, IPV in young women is usually very subtle and common, as it is linked to the romantic characteristics of relationships in this age group. These aspects make young women more vulnerable [16].

Among the barriers related to professionals, the limited training in gender perspective and in caring for adolescents and young people, specifically considering the characteristics of IPV in this particular age group, can be highlighted. This barrier has also been found in other studies conducted in Spain both in health and social services [17–19]. The group of informants in this study have highlighted the importance of having a reference figure with specific training in IPV within the health services, such as primary care, as it improves coordination with internal services within primary care and external services, among others [17]. This study finds that insufficient professional training hinders coordination between different services and can promote secondary victimization of young women. A key aspect for improvement would be to jointly train professionals from different services to enable a more effective coordination between them [40, 41]. As observed in other studies, there seems to be a lack of multidisciplinary work that prevents a comprehensive approach where young women are placed at the center [18]. Thus, another key aspect for improvement would be for the services to include multidisciplinary teams. On the other hand, a weakness in the care provided to young people suffering IPV may be due to the stereotypes professionals have of adolescents, as well as how couple relationships are built in this particular age group, thus making it difficult to detect and care for young women who have experienced IPV. Some professionals may aggravate the situation, leading to secondary victimisation. This is due to their beliefs influenced by gender stereotypes and the minimisation of IPV among young people. As mentioned above, it would be key to address this issue to have professional referrals in the multi-disciplinary team who are dedicated to training others about IPV at all ages [17]. Outside the scope of IPV, adapting services to young people or creating specific resources for this group with trained professionals has beneficial effects [42]. According to the study by Thomée et al., focusing on becoming acquainted with young people and adapting our attitudes towards them is crucial to guarantee a comprehensive care [21]. Key aspects to overcome this barrier would be to specifically train professionals in caring for young women, enabling interpersonal skills adapted to young women to be adopted, such as active listening and empathy, thus generating spaces of trust.

Within the structural barriers of the services, professionals find deficiencies in how these resources are adapted to young women. In particular, they indicate that the infrastructure of the service itself is unwelcoming to young women and even the name of the service is questioned. Therefore, it is important to improve the space itself, not only by promoting more welcoming spaces for young women, but also the dynamics, basing them more on informal activities and sessions as compared to those carried out with adult women. In this way, what Aday & Andersen in 1974 called the “level of integration” of the whole process of resource care is encouraged. In other words, adapting the spaces and dynamics to the young people themselves would allow for continuity in the use of the resources, as they would feel at ease. This continuity is an essential aspect for the use of the services and would integrate the whole care process [43]. Another notable structural barrier is the lack of knowledge and visibility of the services by part of the population. Within education, leading mixed group interventions with adolescents reduces violence due to its primary prevention [35]. In addition, through these interventions, adolescents are made aware of these resources. Likewise, group interventions with young women encourage them to find references and rely on other women who have gone through the same process [44, 45]. There is evidence that group spaces help women connect with their peers and share life experiences, thus leading to a preventive alternative for health promotion. In other words, these self-help groups are health promotion tools for tertiary prevention that aim to help women recover with as few injuries as possible. The exchange of perceptions, ideas, feelings and projects allows some women to come out of their isolation and participate in a listening space, where they can share their experiences, feel understood and become aware of their situation [46, 47].

The professionals who have conducted this study agree that the accessibility problems existing in their services are an important structural barrier, regarding the rigid schedule, the location of some services and the fact that they do not take into account the economic capacity or equal accessibility to the services by young people with different social conditions. According to Takahashi’s framework on determinants of health services use, in order to engage with young girls, it is essential that services are available and accessible, and that they to operate in their same settings, whether it is in community experiences or on social networks [48]. Finally, another structural barrier is the lack of financial resources and adequately trained human resources, leading to some services being saturated and not able to provide quality care to young women. Other studies place more emphasis on the barrier posed by the lack of resources of the women themselves to be able to seek help from these services [28].

The following is a summary of the changes identified that would be necessary to address the barriers perceived by participants (see Table 3).

**Table 3:** Changes that need to be implemented to address the identified problems.

**Changes related to young people**	• Adaptation of services to the needs of young people (timetables, location and economic capacity). • Creation of specific IPV resources for young people. • Adaptation of resource awareness campaigns to IPV among young people. • Promote support groups among women. • Promote community awareness programmes and IPV prevention programmes in the educational setting. • Take into account the intersectionality of people (migrants, youth, disability, etc.).
**Changes related to professionals**	• Specific training for professionals on IPV and care for young people. • Work in multidisciplinary teams. • Improvement of coordination between professionals with the creation of protocols for action.
**Changes related to resources**	• Improve the infrastructure and dynamics of services to make them more welcoming to young women. • Provide more financial resources to IPV services.

### Limitations and strengths

The purposeful sampling method used could lead to selection bias. It is possible that the selected respondents may have been more sympathetic with the problem of IPV in young women due to their professional experience. Another limitation of the study is that providers offer a second-hand account of young women’s experiences, although their account of their experience and difficulties in providing comprehensive attention to young women is valuable. It would be advisable in the future to address the perspective of young women. The credibility of the study was ensured through the different revisions and contributions made by all authors. In order to ensure reliability, literal quotes from the informants were used, as well as a design with emerging categories and a detailed description of the methodological process. At the same time, the use of an inductive approach with a coding process helps to endorse the results of the study. Lastly, by describing the study context, the results of the study can be more easily adapted to other similar contexts [49].

## Conclusions

This study has identified barriers and aspects for improvement which will enable changes that need to be implemented, as well as new interventions and strategies aimed both at the services to prevent and respond to IPV and at the group of professionals who work within those services. These changes would improve the care provided to young women who belong to a group with a specific set of characteristics who can be helped, as explained throughout this manuscript. The scientific evidence generated needs to be applied in order to improve both the services and the training professionals receive in order to achieve a better response from the system to the social problem of IPV against young women; a problem that conditions and will condition the well-being of future generations of women and their environment.

## Supporting information

S1 Table COREQ checklist. Consolidated criteria for reporting qualitative research.
(DOCX)

S2 Table Summary of the script used in the interviews of the professionals. (DOCX)
