# Supplementary material for: Accessibility of intimate partner violence-related services for young women in Spain. Qualitative study on professionals’ perspectives
Source: PLoS One. 2024 Apr 4;19(4):e0297886. doi: 10.1371/journal.pone.0297886 (PMC10994297; doi:10.1371/journal.pone.0297886)
Supplement: S1 Table — Consolidated criteria for reporting qualitative research. (DOCX) [file pone.0297886.s001.docx]

**S1 Table COREQ CHECKLIST. Consolidated criteria for reporting qualitative research**

| **No. Item** | **Guide questions/description** | **Reported** |
| --- | --- | --- |
| **Domain 1: Research team an reﬂexivity** | | |
| 1. Inter viewer/facilitator | Which author/s conducted the  interview? | *The interviews were conducted by ECT as part of the research team with training and experience in qualitative research methodology. Moreover, she is an equality agent (a qualified professional figure in the field of equality policies and violence against women).* |
| 2. Credentials | What were the researcher’s credentials? |  |
| 3. Occupation | What was their occupation at the time of the study? |  |
| 4. Gender | Was the researcher male or female? | *The interviews were conducted by one female researcher, ECT.* |
| 5. Experience and training | What experience or training did the researcher have? | *The interviewer has sufficient training and experience in qualitative research methodology.* |
| 6. Relationship with participants established | Was a relationship established prior to study commencement? | *No* |
| 7. Participant knowledge  of the interviewer | What did the participants know about  the researcher? | *The interviewer was also the one who contacted the participants for the recruitment of the sample. Therefore, they were informed by her about the project, the research leader, the organization responsible for the project, its scope, and the objectives of the study.* |
| 8. Interviewer  characteristics | What characteristics were reported  about the inter viewer/facilitator? |  |
| ***Domain 2: study design*** | | |
| 9. Methodological  orientation and Theory | What methodological orientation was  stated to underpin the study? | *Content analysis* |
| 10. Sampling | How were participants selected? | *The strategy for the identification, selection and recruitment of participants was as follows. Selection of potential participants based on prior knowledge (ECT). Discussion with the research team. Identification of new participants. Telephone contact with each informant (ECT). Formal invitation by e-mail, after informing their superiors if necessary. (ECT, BSB).* |
| 11. Method of approach | How were participants approached? | ECT contacted the 17 potential professionals by telephone. They agreed to participate in the study and were formally invited via an email, that contextualised the study and explained the aim of the interview. |
| 12. Sample size | How many participants were in the study? | *The 17 professionals (14 women and 3 men) of different occupations and different levels of administration* |
| 13. Non-­‐participation | How many people refused to participate or dropped out? Reasons? | *All the proposed participants accepted and there were no drop-outs.* |
| 14. Setting of data collection | Where was the data collected? | *The interviews were conducted face-to-face at the participants' place of work.* |
| 15. Presence of non-­‐  participants | Was anyone else present besides the  participants and researchers? | In one interview, two professionals with ECT were present. |
| 16. Description of sample | What are the important characteristics of the sample? | *The 17 professionals (14 women and 3 men) were nurses, a psychiatrist, professionals from the state security forces, psychologists, social workers and those responsible for youth centres or gender violence. In addition, the resources where they worked were divided into the three administrative levels required. (State, regional, municipal and community).* |
| 17. Interview guide | Were questions, prompts, guides provided by the authors? | *This script was elaborated and discussed by all the authors. The interview was validated by the research team. A pilot test was not conducted but the first two interviews were used to see how the questions flowed in each block, which was taken into account for the rest.* |
| 18. Repeat interviews | Were repeat interviews carried out? | *It was necessary to repeat an interview due to a problem with the recording.* |
| 19. Audio/visual recording | Did the research use audio or visual recording to collect the data? | *The audio recorder was used to collect interview data.* |
| 20. Field notes | Were ﬁeld notes made during and/or after the interview? | *The interviewer collected notes during the interview. She also recorded herself after the interview commenting on her experience and perceptions.* |
| 21. Duration | What was the duration of the interviews? | *All interviews were recorded and lasted between 45 and 90 minutes, with an average of 1 hour.* |
| 22. Data saturation | Was data saturation discussed? | *The research team considered that data saturation was achieved when latest interviews did not generate new additional information*. |
| 23. Transcripts returned | Were transcripts returned to  participants for comment and/or correction? | *No* |
| **Domain 3: analysis and ﬁndings** | | |
| 24. Number of data coders | How many data coders coded the data? | *One analyst from the research team (ECT with the support of LOG)* |
| 25. Description of the  coding tree | Did authors provide a description of the  coding tree? | *No, because the coding tree was not used for analysis* |
| 26. Derivation of themes | Were themes identiﬁed in advance or derived from the data? | *No previous themes were identified in the analysis. Categories emerged by grouping the transcript data according to their similarity to each other.* |
| 27. Software | What software, if applicable, was used to manage the data? | *No software was used* |
| 28. Participant checking | Did participants provide feedback on the ﬁndings? | *Because of the overload work of the professionals from IPV-response services we decide not to recontact them, so feedback was not collected.* |
| 29. Quotations presented | Were participant quotations presented to illustrate the themes/ﬁndings? Was  each quotation identiﬁed? | *Identified quotations are added in the manuscript in results section* |
| 30. Data and ﬁndings consistent | Was there consistency between the data presented and the ﬁndings? | *Yes* |
| 31. Clarity of major themes | Were major themes clearly presented in the ﬁndings? | Yes |
| 32. Clarity of minor  themes | Is there a description of diverse cases or discussion of minor themes? | Yes |
