# Supplementary material for: Accessibility of intimate partner violence-related services for young women in Spain. Qualitative study on professionals’ perspectives
Source: PLoS One. 2024 Apr 4;19(4):e0297886. doi: 10.1371/journal.pone.0297886 (PMC10994297; doi:10.1371/journal.pone.0297886)
Supplement: S2 Table — (DOCX) [file pone.0297886.s002.docx]

| 1: Welcome and introduction. | Contextualisation; Professional career; Link with the intimate partner violence (IPV) intervention and with youth population. |
| --- | --- |
| 2: Current situation of IPV in young women. | Opinion about IPV, specifically in a couple context in young women; differences in IPV in youth/adolescents and older women, and how they influence your daily work. |
| 3: Approaching IPV from the professional practice. | Perceptions regarding the knowledge about the services by young women and your response when faced with an IPV situation; Perceptions on the access and use of social health services and formal resources by young women; What are the motivations of young women to make use, or not, of these type of services; Contribution of the service to modify the violence situation suffered by the young woman; System for case follow-up or implementation; Assessment of other formal resources available for IPV and your work with young women. |
| 4: Difficulties in accessing the services designed to tackle IPV in young women | Main barriers for young women to access and use the formal resources for IPV; Professional difficulties to effectively manage IPV in young women; Perceptions regarding whether the young women find the services “friendly”; Possible structural barriers or barriers related to training or awareness of gender equality and using an intersectional approach; Service adaptations that are needed to help young women, and factors that facilitate quality care. |
| 5: Proposals for improvement | Reflection on proposals for improvement; Other considerations. |

# S2 Table. Summary of the script used in the interviews of the professionals.
